# ﻿*Cytosporalongdensis* sp. nov. and *C.sinensis* (Diaporthales, Valsaceae) associated with *Populusalba* subsp. *pyramidalis* canker and dieback in China

**DOI:** 10.3897/mycokeys.118.152880

**Published:** 2025-06-13

**Authors:** Jieqiong Li, Canting Li, Ganlin Wang, Liangliang Zhu, Lili Huang

**Affiliations:** 1 State Key Laboratory for Crop Stress Resistance and High-Efﬁciency Production, College of Forestry, Northwest A&F University, Yangling, Shaanxi 712100, China Northwest A&F University Shaanxi China; 2 State Key Laboratory for Crop Stress Resistance and High-Efﬁciency Production, College of Plant Protection, Northwest A&F University, Yangling, Shaanxi 712100, China Northwest A&F University Shaanxi China

**Keywords:** Host preference, morphological characteristics, multi-locus phylogeny, pathogenicity, *Valsa*

## Abstract

Poplar is an important afforestation tree species globally and is widely cultivated in northern China. During a small-scale local disease survey in Ningxia, China, canker and dieback symptoms were observed in Populusalbasubsp.pyramidalis trees. The aim of this study was to identify the isolates associated with the symptoms observed and evaluate their pathogenicity on Populusalbasubsp.pyramidalis, *Malusdomestica* cv. ‘Fuji’ and *Pyrusbretschneideri*. Based on multi-gene phylogenetic analyses (ITS, *act*, *rpb2*, *tef1-α*, and *tub2*) and morphological comparisons, two *Cytospora* species were identified: *C.sinensis* and a novel species described here as *C.longdensis***sp. nov.** Pathogenicity assays confirmed both species were pathogenic to Populusalbasubsp.pyramidalis, with *C.sinensis* exhibiting significantly stronger virulence than *C.longdensis* (p < 0.05). In contrast, their pathogenicity on *Malusdomestica* cv. ‘Fuji’ and *Pyrusbretschneideri* was negligible, indicating their host preference for Populusalbasubsp.pyramidalis. This study highlights the host-specific adaptation of *Cytospora* species and provides critical insights for managing Populusalbasubsp.pyramidalis canker and dieback diseases.

## ﻿Introduction

Poplars (*Populus* spp.) are among the most widely used afforestation and greening species in China due to their strong ecological adaptability, rapid growth, and high-yield timber. Their total plantation area in China exceeds 7.57 million hectares, of which 4.45 million hectares are timber forest (Forest Resources Management Division 2018; Lin et al. 2023). However, canker and dieback caused by some *Cytospora* species are prevalent in *Populus* stands, particularly in northern China, resulting in significant ecological and economic losses (Zhuang 2005; Fan et al. 2020; Lin et al. 2023).

The genus *Cytospora* (Cytosporaceae, Diaporthales) comprises many important plant pathogens, such as *Cytosporachrysosperma*, *C.leucosperma*, and *C.mali* (=*Valsamali*), etc. These pathogens are associated with canker and dieback disease in herbaceous and woody plants worldwide (Adams et al. 2005; Feng et al. 2023; Lin et al. 2024). The pathogens usually invade the host tissues through small cracks, wounds, and natural openings on the bark surface. Later, they can colonize and decompose the cambium and penetrate extensively into the phloem and xylem, causing cortical tissue rot, slight depressions, cracks, and discoloration (Feng et al. 2023; Azizi et al. 2024). Finally, cankers and diebacks were formed on the infected plants, which can lead to host plant mortality in severe cases (Pan et al. 2021; Feng et al. 2023; Azizi et al. 2024).

Species of *Cytospora* were often known by their asexual morph: pycnidia stromata submerged in bark, with single or labyrinthiform cavities visible in tangential section, with or without black conceptacles, filamentous conidiophores, phialidic conidiogenous cells, and allantoid hyaline spores (Adams et al. 2005; Lawrence et al. 2018; Fan et al. 2020; Lin et al. 2024). This is largely due to the fact that the asexual state is the one most commonly encountered on diseased plants in nature. In the sexual state, the ascomata are immersed in bark tissues with erumpent pseudostromata; ellipsoid to clavate asci contain four, eight, or more elongate-allantoid hyaline ascospores (Spielman 1985; Adams et al. 2005; Lin et al. 2024).

Traditionally, species of *Cytospora* were identified based on their morphological characteristics and host associations (Donk 1964; Spielman 1985; Adams et al. 2005). However, the morphological variation between some species was complicated. The spore size of the same species could vary depending on the host plant and climatic conditions (Fan et al. 2020; Lin et al. 2023, 2024). This led to incorrect species identification and the fact that cryptic species were often overlooked (Norphanphoun et al. 2017; Fan et al. 2020).

The emergence of DNA sequencing technology has greatly enhanced our scientific capacity to recognize cryptic species and identify unknown species. A combination of five gene regions, including the nuclear rDNA internal transcribed spacer region (ITS), actin (*act*), RNA polymerase II subunit (*rpb2*), translation elongation factor 1-alpha (*tef1-α*), and β-tubulin (*tub2*), has been found to provide stable and reliable resolution for the identification of *Cytospora* species (Norphanphoun et al. 2017, 2018; Lawrence et al. 2018; Fan et al. 2020; Lin et al. 2024). Based on the DNA sequence analysis of these five gene regions, Lin et al. (2024) recently identified 399 *Cytospora* isolates from 32 countries and proposed 44 novel species.

To date, about 36 species of *Cytospora* spp. have been reported on poplar trees worldwide, of which 24 are found in China, including *C.ailanthicola*, *C.alba*, *C.atrocirrhata*, *C.beijingensis*, *C.chrysosperma*, *C.davidiana*, *C.deqinensis*, *C.diqingensis*, *C.donglingensis*, *C.eastringensis*, *C.fugax*, *C.hoffmannii* (a synonym of *C.nivea* and *C.paratranslucens*), *C.kantschavelii*, *C.lijiangensis*, *C.palmoides*, *C.populi*, *C.pseudochrysosperma*, *C.sanbaensis*, *C.shangrilaensis*, *C.sinensis*, *C.sophoriopsis*, *C.translucens*, *C.tritici*, and *C.yuduensis* (Zhuang 2005; Zhang et al. 2012, 2013; Wang et al. 2013, 2015; Fan et al. 2020; Lin et al. 2023, 2024).

During our recent investigations in the Ningxia Hui Autonomous Region, China, a disease exhibiting symptoms of trunk, crown, and collar canker, as well as branch dieback, was observed in a stand of approximately fifty 10-year-old Populusalbasubsp.pyramidalis trees. The aim of this study was to (1) identify the isolates associated with the canker and dieback disease and (2) evaluate the pathogenicity of the isolates on *Populusalbasubsp.pyramidalis* (Bunge) W. Wettst, *Malusdomestica* (Suckow) Borkh. cv. ‘Fuji’ and *Pyrusbretschneideri* Rehd.

## ﻿Sample collection and fungal isolation

During 2022–2023, a disease survey was conducted in a 700-m^2^ stand of approximately fifty 10-year-old Populusalbasubsp.pyramidalis trees in Longde County, Ningxia Hui Autonomous Region, China. Three trees exhibiting canker and dieback symptoms were examined. Twigs, branches, and trunk cortex segments with obvious fruiting bodies were sampled, packed in clean paper bags, and transferred to the laboratory for further research.

Under a dissecting microscope (Olympus SZX16, Tokyo, Japan), conidiomata and ascomata formed under the bark on the specimens were sectioned using a sterile scalpel. Mucoid spore masses were transferred directly to 2% malt extract agar (MEA: 20 g malt extract powder, 20 g agar powder, distilled water to complete 1000 mL) using sterile needles. After one day of incubation at 25 °C, a single hyphal tip from each culture was transferred to new MEA plates and incubated at 25 °C for seven days. The herbarium specimens and cultures were deposited in the culture collection of State Key Laboratory for Crop Stress Resistance and High-Efﬁciency Production, Northwest A&F University (NWAFU).

### ﻿DNA extraction, PCR amplification, and sequencing

The total genomic DNA was extracted from one-week-old cultures using Prepman™ Ultra Sample Preparation Reagent (Thermo Fisher Scientific, Waltham, MA, USA) following the protocol described by Duong et al. (2012). Five gene regions, including ITS, *act*, *rpb2*, *tef1-α*, and *tub2*, were amplified using the protocol described by Fan et al. (2020). Amplicons were sequenced in both directions by Sangon Biotech Company Limited, Shanghai, China. The sequences obtained were assembled using Geneious v. 7.0 (Kearse et al. 2012) and subjected to BLASTn searches to preliminarily identify their classification. Genotypes of the isolates were determined based on the sequences of the ITS, *act*, *rpb2*, *tef1-α*, and *tub2* gene regions. Isolates from three distinct trees, showing different morphological characteristics and genotypes, were selected for phylogenetic analyses and pathogenicity tests. All sequences generated in this study were deposited in GenBank (Table 1).

**Table 1.** Strains of *Cytospora* used in the phylogenetic analyses in this study.

Name	Strain	Host	Origin	GenBank accession numbers				
				ITS	*act*	*rpb2*	*tef1-α*	*tub2*
*C.ailanthicola*	CFCC 58226	*Populus×canadensis*	Yunnan, China	PP988715	PQ074583	PQ074896	PQ074255	PQ075213
*C.ailanthicola*	CFCC 89970^T^	*Ailanthusaltissima*	Ningxia, China	MH933618	MH933526	MH933592	MH933494	MH933565
*C.azerbaijanica*	IRAN 4201C^T^	*Malusdomestica*	Urmia, Iran	MW295526	MZ014513	MW824360	MW394147	NA
*C.azerbaijanica*	IRAN 4627C	*Malusdomestica*	Miandoab, Iran	OM368650	NA	NA	OM372512	NA
*C.chrysosperma*	CBS 134.25	*Fraxinusamericana*	NA	PP988775	PQ074628	PQ074944	PQ074306	PQ075257
*C.chrysosperma*	CBS 197.50^T^	*Populustremula*	UK	PP988777	PQ074630	PQ074946	PQ074308	PQ075259
*C.cinnamomea*	CFCC 53178^T^	*Prunusarmeniaca*	Xinjiang, China	MK673054	MK673024	NA	NA	MK672970
*C.deqinensis*	CFCC 58467^T^	*Populushaoana*	Yunnan, China	PP988795	PQ074646	PQ074961	PQ074325	PQ075275
*C.deqinensis*	CFCC 58468	*Populushaoana*	Yunnan, China	PP988796	PQ074647	PQ074962	PQ074326	PQ075276
*C.donglingensis*	CFCC 53159^T^	*Platycladusorientalis*	Beijing, China	MW418412	MW422903	MW422915	MW422927	MW422939
*C.donglingensis*	CFCC 58491	*Populus×canadensis*	Yunnan, China	PP988816	PQ074659	PQ074978	PQ074344	PQ075292
*C.eastringensis*	CFCC 58222^T^	*Populusadenopoda*	Yunnan, China	PP988818	NA	PQ074980	PQ074346	NA
*C.euonymina*	CFCC 89993^T^	*Euonymuskiautschovicus*	Shanxi, China	MH933630	MH933537	MH933600	MH933505	MH933590
*C.guyuanensis*	CFCC 55855^T^	*Salix* sp.	Ningxia, China	PP988853	NA	PQ075011	PQ074378	PQ075323
*C.guyuanensis*	CFCC 56037^T^	*Salix* sp.	Ningxia, China	PP988854	NA	PQ075012	PQ074379	PQ075324
*C.hejingensis*	C3479	*Salix* sp.	Xinjiang, China	PP060456	PP059658	PP059664	PP059668	PP059674
*C.hejingensis*	CFCC 59571^T^	*Salix* sp.	Xinjiang, China	PP060455	PP059657	PP059663	PP059667	PP059673
*C.hippophaopsis*	XJAU 1378^T^	*Malussieversii*	Xinjiang, China	PP965505	PP957863	PP957870	PP957877	PP957884
*C.hippophaopsis*	XJAU 1379	*Malussieversii*	Xinjiang, China	PP965506	PP957864	PP957871	PP957878	PP957885
*C.joaquinensis*	CBS 144235^T^	*Populusdeltoides*	California, USA	MG971895	MG972044	NA	MG971605	OP079911
*C.kantschavelii*	CFCC 58213	*Populus×canadensis*	Yunnan, China	PP988875	PQ074706	PQ075028	PQ074398	PQ075340
*C.kantschavelii*	MFLUCC 15-0857^T^	Populus×sibirica	Russia	KY417738	KY417704	KY417806	NA	NA
*C.lauricola*	CFCC 58193^T^	*Laurusnobilis*	Yunnan, China	PP988881	PQ074711	PQ075033	PQ074404	PQ075345
*C.lauricola*	CFCC 58221^T^	*Laurusnobilis*	Yunnan, China	PP988883	PQ074713	PQ075034	PQ074406	PQ075346
***C.longdensis***	**D116^T^**	**Populusalbasubsp.pyramidalis**	**Ningxia, China**	**PV241400**	**PV246004**	**PV256729**	**PV256735**	**PV256741**
***C.longdensis***	**D117**	**Populusalbasubsp.pyramidalis**	**Ningxia, China**	**PV241401**	**PV246005**	**PV256730**	**PV256736**	**PV256742**
***C.longdensis***	**D118**	**Populusalbasubsp.pyramidalis**	**Ningxia, China**	**PV241402**	**PV246006**	**PV256731**	**PV256737**	**PV256743**
*C.lhasaensis*	CFCC 58706^T^	*Salix* sp.	Tibet, China	PP988902	PQ074732	PQ075052	PQ074425	PQ075365
*C.lijiangensis*	CFCC 58483^T^	*Populus×canadensis*	Yunnan, China	PP988905	PQ074735	PQ075054	PQ074428	NA
*C.lijiangensis*	CFCC 89999	*Euonymuskiautschovicus*	Shanxi, China	MH933631	MH933538	MH933601	MH933506	MH933591
*C.longiostiolata*	MFLUCC 16-0628	Salix×fragilis	Russia	KY417734	KY417700	KY417802	NA	NA
*C.melnikii*	CFCC 89984	*Rhustyphina*	Xinjiang, China	MH933644	MH933551	MH933609	MH933515	MH933580
*C.melnikii*	MFLUCC 15-0851^T^	*Malusdomestica*	Russia	KY417735	KY417701	KY417803	NA	NA
*C.michailidesia*	IRAN 4409C^T^	*Malusdomestica*	Oshnavieh, Iran	OP020558	OP018921	OP018909	NA	NA
*C.michailidesia*	IRAN 4410C	*Malusdomestica*	Oshnavieh, Iran	OP020559	OP018922	OP018910	NA	NA
*C.miyandoabensis*	IRAN 4405C^T^	*Malusdomestica*	Miyandoab, Iran	OP377064	OP432090	OP432087	NA	NA
*C.miyandoabensis*	IRAN 4406C	*Malusdomestica*	Miyandoab, Iran	OP377065	OP432091	OP432088	NA	NA
*C.nitschkeana*	CBS 118.22^T^	*Salixalba*	Netherlands	PP988924	PQ074752	PQ075071	PQ074445	PQ075385
*C.nitschkeana*	CBS 195.42	NA	Switzerland	PP988925	PQ074753	PQ075072	PQ074446	PQ075386
*C.nobilis*	CFCC 58227	*Laurusnobilis*	Yunnan, China	PP988928	PQ074756	PQ075075	PQ074449	PQ075389
*C.nobilis*	CFCC 58228	*Laurusnobilis*	Yunnan, China	PP988929	PQ074757	PQ075076	PQ074450	PQ075390
*C.paracinnamomea*	CFCC 55453^T^	*Salixmatsudana*	Gansu, China	MZ702594	OK303456	OK303515	OK303576	OK303643
*C.paracinnamomea*	CFCC 55455^T^	*Salixmatsudana*	Gansu, China	MZ702598	OK303460	OK303519	OK303580	OK303647
*C.pseudochrysosperma*	CFCC 89981^T^	*Populusalbasubsp.pyramidalis*	Gansu, China	MH933625	MH933533	MH933597	MH933501	MH933568
*C.pseudochrysosperma*	CFCC 89982	*Ulmuspumila*	Tibet, China	KP281261	KP310835	KU710952	KP310848	KP310818
*C.rostrata*	CFCC 89909^T^	*Salixcupularis*	Gansu, China	KR045643	KU711009	KU710974	NA	NA
*C.rostrata*	CFCC 89910	*Salixcupularis*	Gansu, China	KR045644	KU711010	KU710975	KU710933	NA
*C.salicacearum*	MFLUCC 15-0509^T^	*Salixalba*	Russia	KY417746	KY417712	KY417814	NA	NA
*C.salicacearum*	MFLUCC 15-0861	Salix×fragilis	Russia	KY417745	KY417711	KY417813	NA	NA
*C.salicicola*	MFLUCC 14-1052^T^	*Salixalba*	Russia	KU982636	KU982637	NA	NA	NA
*C.salicicola*	MFLUCC 15-0866	*Salix* sp.	Thailand	KY417749	KY417715	KY417817	NA	NA
*C.salicina*	CBS 141629	*Vitisvinifera*	Iran	PP988980	PQ074803	PQ075121	PQ074496	PQ075434
*C.salicina*	MFLUCC 15-0862^T^	*Salixalba*	Russia	KY417750	KY417716	KY417818	NA	NA
*C.schulzeri*	CBS 118570	*Malussylvestris*	Michigan, USA	PP988985	PQ074808	PQ075126	PQ074500	PQ075439
*C.schulzeri*	MFLUCC 15-0507^T^	*Malusdomestica*	Russia	KY417740	KY417706	KY417808	NA	NA
*C.sinensis*	CFCC 58224^T^	*Populussimonii*	Gansu, China	PP988994	PQ074817	PQ075135	PQ074509	PQ075448
*C.sinensis*	CFCC 58235^T^	*Populussimonii*	Gansu, China	PP988997	PQ074820	PQ075138	PQ074512	PQ075451
***C.sinensis***	**D180**	**Populusalbasubsp.pyramidalis**	**Ningxia, China**	**PV241403**	**PV246007**	**PV256732**	**PV256738**	**PV256744**
***C.sinensis***	**D182**	**Populusalbasubsp.pyramidalis**	**Ningxia, China**	**PV241404**	**PV246008**	**PV256733**	**PV256739**	**PV256745**
***C.sinensis***	**D184**	**Populusalbasubsp.pyramidalis**	**Ningxia, China**	**PV241405**	**PV246009**	**PV256734**	**PV256740**	**PV256746**
*C.sophoriopsis*	CFCC 58464	*Populusszechuanica*	Yunnan, China	PP989009	PQ074831	PQ075150	PQ074524	PQ075461
*C.sophoriopsis*	CFCC 89600^T^	*Styphnolobiumjaponicum*	Gansu, China	KR045623	KU710992	KU710951	KU710915	KP310817
*C.suecica*	CBS 450.51^T^	*Populustremula*	Sweden	PP989015	PQ074834	PQ075156	PQ074530	PQ075467
*C.tritici*	CBS 141625	*Prunus* sp.	Iran	PP989040	PQ074858	PQ075177	PQ074551	PQ075488
*C.tritici*	IRAN 4198C	*Malusdomestica*	Iran	MW295523	MZ014510	MW824357	MW394144	NA
*C.yakimana*	CBS 149297^T^	*Vitisvinifera*	USA	OM976602	ON012555	ON045093	ON012569	ON086750
*C.yakimana*	CBS 149298	*Vitisvinifera*	USA	OM976603	ON012556	ON045094	ON012570	ON086751
*C.yinchuanensis*	CFCC 50040^T^	*Malusdomestica*	Ningxia, China	KR045649	KU711013	KU710980	KU710936	KR045690
*C.yinchuanensis*	CFCC 50042^T^	*Malusasiatica*	Gansu, China	KR045650	KU711014	KU710981	KU710937	KR045691
*Diaporthevaccinii*	CBS 160.32	*Vacciniummacrocarpon*	USA	KC343228	JQ807297	NA	KC343954	KC344196

**Note**: Type strains are marked with “T”; “NA” represents not available; Isolates from the present study are typed in bold.

### ﻿Phylogenetic analyses

The referenced sequences of phylogenetically related *Cytospora* species were downloaded from NCBI (http://www.ncbi.nlm.nih.gov). *Diaporthevaccinii* (CBS 160.32) was selected as the outgroup taxon (Lin et al. 2024). Sequences generated from this study and those from NCBI were aligned using the MAFFT v. 7 online version (https://mafft.cbrc.jp/alignment/server/) and manually edited in MEGA v. 6.0 (Tamura et al. 2013).

Maximum likelihood (ML) and Bayesian inference (BI) methods were used for phylogenetic analyses on the CIPRES Science Gateway platform (Miller et al. 2010). The sequence datasets for the five individual gene regions were initially analyzed separately to determine the phylogenetic relatedness of all isolates. Subsequently, a multi-gene phylogenetic analysis was conducted for the combined dataset of the five gene regions. The GTR+G+I substitution model and 1000 bootstrap replicates were set for ML analysis in RAxML v. 8.2.12 (Stamatakis 2014). For BI phylogenetic analysis, the appropriate substitution model was generated for each partitioned locus using jModeltest v. 2.1.5 (Guindon and Gascuel 2003; Darriba et al. 2012). Four Markov Chain Monte Carlo (MCMC) chains for 1,000,000 generations were employed for BI analysis in MrBayes v. 3.2.7 (Ronquist et al. 2012). The resultant trees were visualized in FigTree v. 1.4.4 and edited using Adobe Illustrator 2020.

### ﻿Morphology

To induce sporulation, cultures were grown on 2% water agar (WA) plates containing one-year-old, twice-autoclaved poplar shoots on the surface and incubated at room temperature under near-ultraviolet light (12-h photoperiod). The fungal was examined weekly. The fruiting bodies were sectioned (tangentially and vertically) using sterile razor blades and photographed under an Olympus SZX16 stereomicroscope (Tokyo, Japan). The micro-morphological structures were mounted in a drop of 85% lactic acid (Liu et al. 2020) on microscope slides and examined under an Olympus BX53 microscope (Tokyo, Japan). At least 30 replicate measurements were made for each taxonomic character by cellSens imaging software (Olympus, Tokyo, Japan). Colony characteristics were examined on PDA plates after growth at 25 °C in the dark for seven days. Colony colors were described according to the color charts of Rayner (1970).

### ﻿Pathogenicity tests

Healthy one-year-old branches (approximately 2 cm in diameter) of Populusalbasubsp.pyramidalis, *Malusdomestica* cv. ‘Fuji’ and *Pyrusbretschneideri* were collected from the cultivar conservation nursery of Northwest A&F University (34°15'41.7"N, 108°3'33.3"E). The collected branches were surface-disinfected in a 3% sodium hypochlorite solution for 90 s, rinsed three times with sterile distilled water, and dried at room temperature. The cut ends of the branches (approximately 50 cm long) were sealed with paraffin to minimize desiccation.

A sterilized cork borer (5 mm in diameter) was used to create a 2 mm deep wound in the center of each branch. Five mm in diameter mycelial plugs were excised from a 3-day-old colony of each selected isolate of *Cytospora* species identified in this study and placed onto the branch wound. The inoculated sites were covered with sterile, moistened cotton balls and wrapped with masking tape to maintain humidity. Sterile MEA served as the negative control, and *C.mali* 03-8, a pathogen causing Apple Valsa Canker from our laboratory culture collection, was designated as the positive control. Three replicates were conducted for each isolate and control, respectively. All of them were incubated in humid chambers at room temperature (25 ± 2 °C) under natural light conditions. Lesion sizes around the inoculation sites were measured on the fifth and tenth days post-inoculation. To fulfill Koch’s postulates, the fungi were re-isolated from the lesion margins and identified based on colony morphology. The experiment was replicated three times following the same protocol. The data were analyzed using one-way analysis of variance (ANOVA) and Tukey’s multiple comparison test (p < 0.05) in IBM SPSS Statistics v. 22 (IBM Corp., USA).

## ﻿Results

### ﻿Isolates and sequencing

A total of nine isolates with typical morphological characteristics of *Cytospora* species were obtained. BLASTn searches and sequence alignments for the ITS, *act*, *rpb2*, *tef1-α*, and *tub2* gene regions confirmed that the nine isolates belong to the genus *Cytospora* and represent three genotypes. Based on the origin details (specific tree and tissue, collection date), morphological characteristics, and genotypes of the isolates, six isolates were selected for further study (Table 1).

### ﻿Phylogenetic analyses

Based on the comparisons of phylogenetic tree topologies for each of the five gene regions, sequences of 63 strains representing 35 *Cytospora* species (Table 1), closely related to the species collected in this study, were selected for multi-gene phylogenetic analyses. The dataset of combined ITS, *act*, *rpb2*, *tef1-α*, and *tub2* sequence alignments comprised 2478 characters, including 475 characters for ITS, 252 characters for *act*, 644 characters for *rpb2*, 619 characters for *tef1-α*, and 488 characters for *tub2*.

For ML analysis, the matrix had 1024 distinct alignment patterns, with 25.07% gaps or undetermined characters. The final ML optimization likelihood value of the best-scoring tree was -14539.91. The estimated base frequencies were as follows: A = 0.250442, C = 0.289329, G = 0.239478, T = 0.220750; substitution rates: AC = 1.420694, AG = 3.784817, AT = 1.797716, CG = 0.974888, CT = 7.890924, GT = 1.000000; and the gamma distribution shape parameter α = 0.304660. For the BI analysis, the best-fit model of nucleotide substitution for each partitioned locus was as follows: ITS = K80+I+G, *act* = HKY+G, *rpb2* = TrNef+G, *tef1-α* = TrN+G, and *tub2* = HKY+I+G.

**Figure 1.**
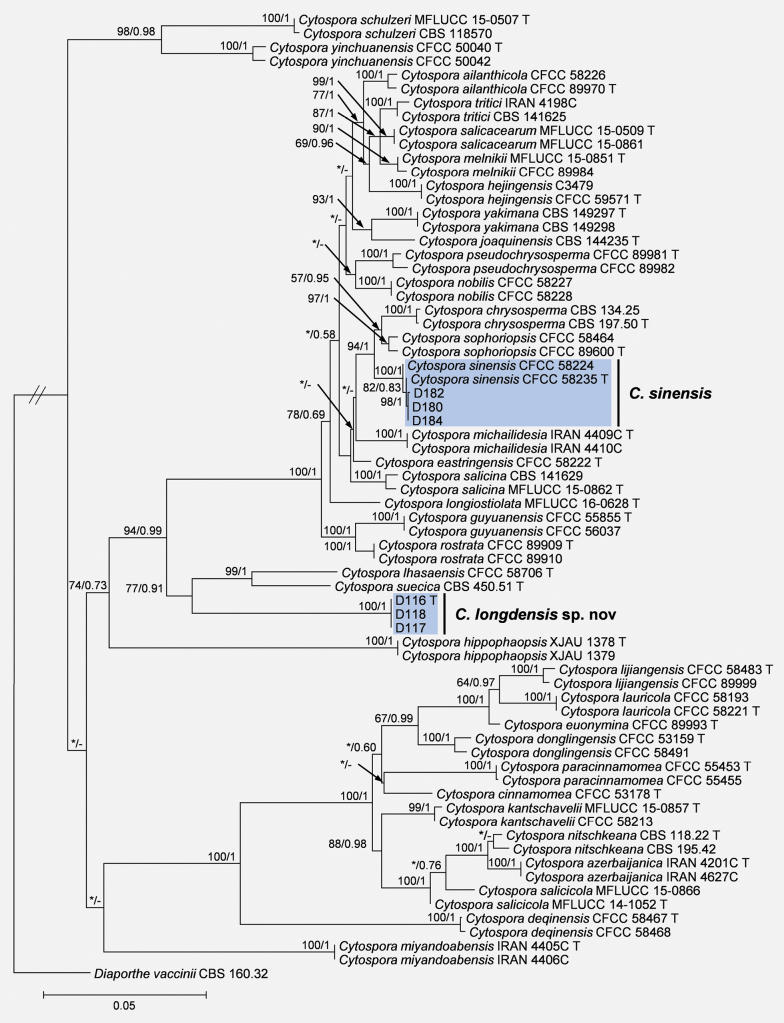
Phylogenetic tree of *Cytospora* based on maximum likelihood analysis of a combined DNA sequence dataset of ITS, *act*, *rpb2*, *tef1-α*, and *tub2* genes. Bootstrap values ≥ 50% (maximum likelihood) and posterior probabilities ≥ 0.5 (Bayesian) are presented at the branches. Values lower than these thresholds are marked with “*”, and absent are marked with “-”. Type materials are marked with “T”. Isolates from this study are highlighted in blue. The tree was rooted to *Diaporthevaccinii* (CBS 160.32).

The tree topology generated from the ML analysis was essentially similar to that produced from the BI analysis. Three isolates (D116, D117, and D118) representing one genotype (genotype I) formed an independent clade distinct from previously known species, supported by high values (ML/BI = 100/1), and were named as *Cytosporalongdensis* sp. nov. (Fig. 1). Isolates D180 (genotype II), D184 (genotype II), and D182 (genotype III), representing two genotypes with only one SNP difference across the five gene regions between genotype II and genotype III, were grouped in the same clade with the ex-type isolate of *C.sinensis* CFCC 58235 (ML/BI = 82/0.83) and were identified as *Cytosporasinensis* (Fig. 1).

### ﻿Taxonomy

#### ﻿ Cytospora longdensis

Taxon classificationFungiDiaporthalesValsaceae

J.Q. Li & L.L. Huang sp. nov.

21291787-CDC5-5936-B0E5-6330464927C5

857641

Fig. 2

##### Etymology.

Named after the locality, Longde County, Guyuan City, where the fungus was first collected.

**Figure 2.**
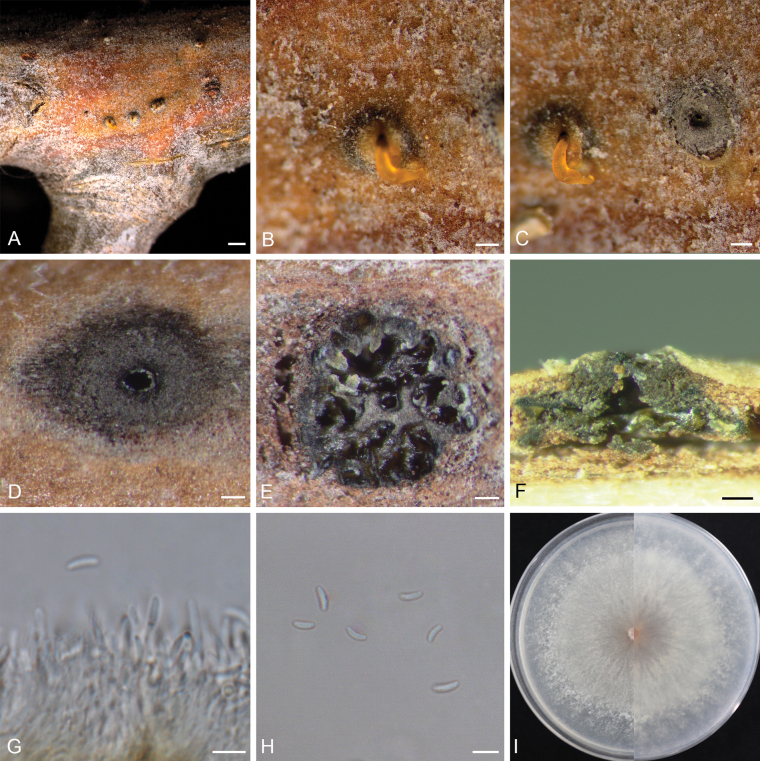
*Cytosporalongdensis* (D 116). **A–C.** View of conidiomata on branch; **D, E.** Transverse section through a conidioma; **F.** Longitudinal section through a conidioma; **G.** Conidiophores, conidiogenous cells, and conidia; **H.** Conidia; **I.** Culture on PDA after five days, front side (left) and back side (right). Scale bars: 500 μm (**A**); 100 μm (**B–F**); 5 μm (**G, H**).

##### Typification.

China • Ningxia Hui Autonomous Region, Guyuan City, Longde County, Shatang Town, 35°35'12.5"N, 106°7'7.4"E, on cankered and diebacked branches of Populusalbasubsp.pyramidalis, 30 July 2022, J.Q. Li & L.L. Huang (holotype: NWAFU H116, ex-holotype culture: D116).

##### Description.

***Sexual morph***: not observed. ***Asexual morph***: Conidiomata immersed or semi-immersed in bark, discoid, with multiple locules. Conceptacle absent. Ectostromatic disc greenish black to black, circular to ovoid, (296–)351–508(–603) μm in diameter, with one ostiole per disc. Ostiole black, circular to ovoid, (42–)52–87(–105) μm in diameter. Locules numerous, subdivided by invaginations with common walls. Conidiophores hyaline, thin-walled, unbranched, or occasionally branched at the bases, 15.1–26.3 × 1.0–2.1 μm (av. = 18.9 ± 2.9 × 1.4 ± 0.3 μm, n = 30). Conidiogenous cells enteroblastic, phialidic, subcylindrical to cylindrical. Conidia hyaline, smooth, allantoid, aseptate, 4.2–5.7 × 1.6–2.2 μm (av. = 5.0 ± 0.4 × 1.8 ± 0.2 μm, n = 50).

##### Culture characteristics.

Colonies at 25 °C on PDA are initially white, flat, reaching 4.7 cm in diameter after three days, becoming buff, and fully covering the 9.0 cm Petri dish after seven days.

##### Additional materials examined.

China • Ningxia Hui Autonomous Region, Guyuan City, Longde County, Shatang Town, 35°35'12.5"N, 106°7'7.4"E, on cankered branches of Populusalbasubsp.pyramidalis, 30 July 2022, J.Q. Li & L.L. Huang (NWAFU H117, culture: D117; NWAFU H118, culture: D118).

##### Notes.

*Cytosporalongdensis* is phylogenetically closely related to *C.suecica* and *C.lhasaensis*. However, it is distinct from them by producing larger conidia, i.e., 4.2–5.7 × 1.6–2.2 μm in *C.longdensis* vs. 0.5–2 × 0.5–1 μm in *C.suecica* and 3.5–5 × 1–1.5 μm in *C.lhasaensis* (Lin et al. 2024). Additionally, it differs from *C.suecica* (CBS 450.51) and *C.lhasaensis* (CFCC 58706) in SNPs for the five gene region sequences (*C.longdensis* vs. *C.suecica*: 23 out of 473 bp in ITS, 28 out of 203 bp in *act*, 26 out of 644 bp in *rpb2*, 70 out of 500 bp in *tef1-α*, and 55 out of 388 bp in *tub2*; *C.longdensis* vs. *C.lhasaensis*: 17 out of 473 bp in ITS, 19 out of 203 bp in *act*, 25 out of 644 bp in *rpb2*, 75 out of 500 bp in *tef1-α*, and 61 out of 388 bp in *tub2*). Additionally, the multi-gene (ITS, *act*, *rpb2*, *tef1-α*, and *tub2*) phylogram reveals that *C.longdensis* represents an independent clade with high support (ML/BI = 100/1, Fig. 1).

#### ﻿ Cytospora sinensis

Taxon classificationFungiDiaporthalesValsaceae

L. Lin & X.L. Fan, Studies in Mycology 109: 381 (2024)

04C7DF46-9D88-5063-9068-EC58B937141A

Fig. 3

##### Description.

***Sexual morph***: not observed. ***Asexual morph***: Conidiomata immersed in bark, erumpent when mature, discoid, with multiple locules. Conceptacle absent. Ectostromatic disc isabelline or greenish black, circular to ovoid, (285–)305–452(–512) μm in diameter, with single ostiole per disc. Ostiole black, circular to ovoid, (38–)45–84(–103) μm in diameter. Locules numerous, divided with shared walls. Conidiophores hyaline, thin-walled, unbranched, or branched at the bases, 12.5–23.2 × 1.2–2.2 μm (av. = 17.6 ± 2.7 × 1.6 ± 0.2 μm, n = 30). Conidiogenous cells enteroblastic, phialidic, subcylindrical to cylindrical. Conidia hyaline, smooth, allantoid, aseptate, 4.5–5.9 × 1.6–2.1 μm (av. = 5.2 ± 0.4 × 1.8 ± 0.1 μm, n = 50).

##### Culture characteristics.

Colonies at 25 °C on PDA are initially white, flat, growing up to 6.2 cm in diameter after three days, becoming olivaceous and completely covering the 9.0 cm Petri dish after five days.

##### Additional materials examined.

China • Ningxia Hui Autonomous Region, Guyuan City, Longde County, Shatang Town, 35°35'12.5"N, 106°7'7.4"E, on cankered and infected branches of Populusalbasubsp.pyramidalis, 30 July 2022, J.Q. Li & L.L. Huang (NWAFU H180, culture: D180; NWAFU H182, culture: D182; NWAFU H184, culture: D184).

##### Notes.

**Figure 3.**
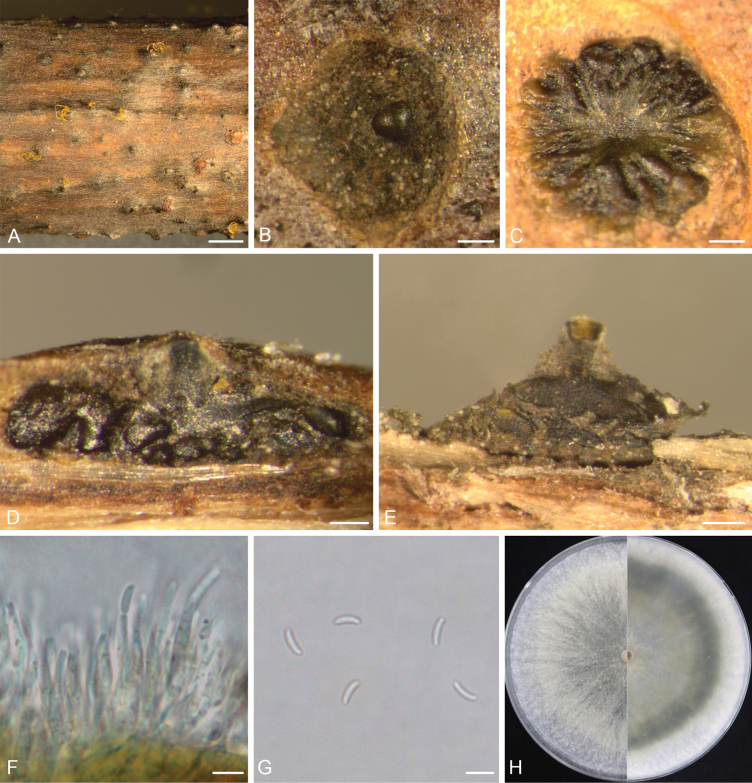
*Cytosporasinensis* (D 184). **A** View of conidiomata on branch; **B, C** Transverse section through a conidioma; **D, E** Longitudinal section through a conidioma; **F** Conidiophores and conidiogenous cells; **G** Conidia; **H** Cultures on PDA after five days, front side (left) and back side (right). Scale bars: 500 μm (**A**); 100 μm (**B, C, E**); 50 μm (**D**); 5 μm (**F, G**).

*Cytosporasinensis* has been reported from *Populus* species in Gansu and Yunnan provinces of China (Lin et al. 2024). In the present study, three isolates causing canker and dieback on Populusalbasubsp.pyramidalis in the Ningxia Hui Autonomous Region were identified as *C.sinensis* based on the phylogenetic topology (Fig. 1) and morphological characteristics consistent with previous descriptions (Fig. 3, Lin et al. 2024).

### ﻿Pathogenicity tests

**Figure 4.**
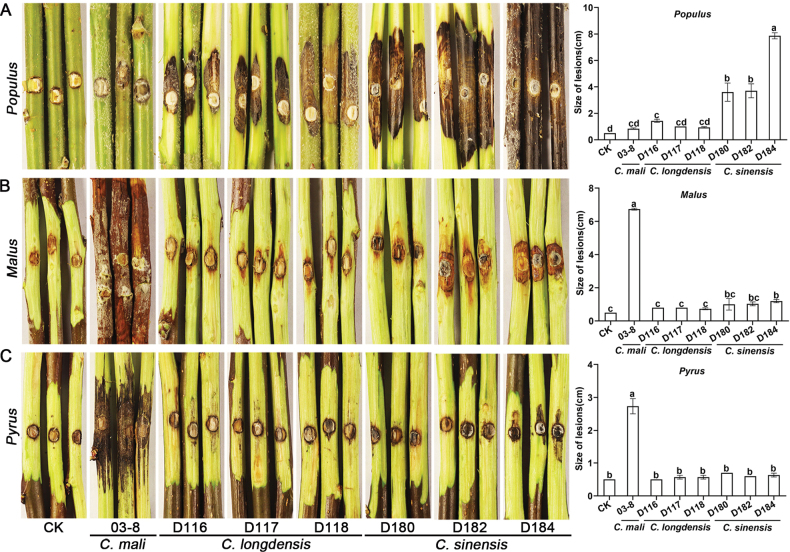
The lesions caused by *Cytospora* isolates on branches of *Populus*, *Malus*, and *Pyrus* five days post-inoculation. **A.***Cytosporalongdensis* (D116, D117, and D118), *C.sinensis* (D180, D182, and D184), and *C.mali* (03-8) on Populusalbasubsp.pyramidalis; **B.** on *Malusdomestica* cv. ‘Fuji’; **C.** and *Pyrusbretschneideri*. Vertical bars represent the standard error of the mean. Different letters above the bars indicate treatment mean values that are significantly different (P = 0.05).

Inoculation of Populusalbasubsp.pyramidalis with conidial masses of *C.longdensis* and *C.sinensis* resulted in the persisting symptoms of canker after five days. No considerable disease symptoms were observed for the negative control (Fig. 4A). Moreover, the lesions on Populusalbasubsp.pyramidalis caused by *C.sinensis* were significantly larger than those caused by *C.longdensis*. However, both species were found to be essentially non-pathogenic to *Malusdomestica* and *Pyrusbretschneideri*, as no clear disease symptoms were observed at the inoculated sites (Fig. 4B, C).

## ﻿Discussion

In this study, we identified two *Cytospora* species, *C.sinensis* and a novel species described as *C.longdensis*, associated with canker and dieback in a stand of approximately fifty 10-year-old Populusalbasubsp.pyramidalis trees in Ningxia, China. Applied pathogenicity assays confirmed that both species were pathogenic to Populusalbasubsp.pyramidalis through nine experimental replicates, with *C.sinensis* exhibiting significantly stronger virulence than *C.longdensis*. In contrast, both species showed negligible pathogenicity on *Malusdomestica* and *Pyrusbretschneideri*.

The co-infection of *C.sinensis* and *C.longdensis* was observed in a small stand of Populusalbasubsp.pyramidalis in Ningxia, China. This finding aligns with previous studies that have reported multiple *Cytospora* species infecting one plant host (Kepley et al. 2015; Lin et al. 2022). As a typical arid to semi-arid region in northwest China, Ningxia has an average annual temperature ranging from 6.9 °C to 11.5 °C (Zhang et al. 2025). The co-infection phenomenon may be linked to environmental stressors, such as drought, frost, and soil salinity, which can weaken host defenses and potentially facilitate co-infections (Adams et al. 2005; Lawrence et al. 2017, 2018; Lin et al. 2024). Understanding the diversity of these fungi is crucial for a possible clarification of their adaptive co-evolution and developing control strategies. Multi-gene phylogenetic analysis combined with morphological comparison provides reliable resolution for identifying cryptic species within the genus *Cytospora* on the same hosts (Norphanphoun et al. 2018; Lin et al. 2024).

Distinct virulence was observed between *C.sinensis* and *C.longdensis*. This suggests that the two species may employ different pathogenic mechanisms or display varying levels of host adaptation (Adams et al. 2005; Sun et al. 2023). *Cytosporasinensis* exhibited stronger virulence, indicating it may play a more dominant role in causing canker and dieback in some *Populus* stands (Lin et al. 2023). In contrast, *C.longdensis* showed weaker virulence, suggesting it may act as a secondary pathogen or require specific environmental conditions to cause significant damage (Adams et al. 2005; Lin et al. 2023). More extensive surveys and further studies are needed to confirm these findings and elucidate the genetic and molecular basis of these virulence differences.

Host preference was observed in this study. Phylogenetic analyses suggested that *C.sinensis* resides in the *C.chrysosperma* complex and is closely related to *C.chrysosperma*. Within the *C.chrysosperma* complex, several species, including *C.chrysosperma* and *C.hoffmannii*, have been identified as pathogens of Salicaceae and Rosaceae (Azizi et al. 2024; Lin et al. 2024). However, *Cytosporasinensis* isolated from *Populus* exhibited limited pathogenicity on *Malus* and *Pyrus*. Its highest virulence towards *Populus* indicates host preference in pathogenicity (Adams et al. 2005; Schulze-Lefert and Panstruga 2011).

*Cytosporasinensis* was recently reported on *Populus* spp. in Gansu and Yunnan provinces, but its pathogenic potential had not yet been confirmed (Lin et al. 2024). Our study fills this gap by confirming the virulence of *C.sinensis* towards Populusalbasubsp.pyramidalis and extending its known geographic distribution to Ningxia, China. The distribution of this species, spanning from northwestern to southwestern China, suggests its strong ecological adaptability. More extensive sampling is required to assess the host range and geographic distribution of *C.sinensis*. Targeted management strategies are needed to control its spread and mitigate associated damages.

This study contributed to the description of a new species, *C.longdensis*, and expanded the known geographical distribution and host-fungus interaction of *C.sinensis* on Populusalbasubsp.pyramidalis. Further studies are required to elucidate the genetic and molecular mechanisms driving their differences in pathogenicity. Moreover, the high virulence and wide distribution range of *C.sinensis* emphasize the urgency of developing targeted control strategies to manage these fungi effectively.

## Supplementary Material

XML Treatment for Cytospora longdensis

XML Treatment for Cytospora sinensis

## ﻿Additional information

### Author ORCIDs

Jieqiong Li https://orcid.org/0000-0001-6982-150X

Lili Huang https://orcid.org/0000-0002-7085-7646

### Data availability

All of the data that support the findings of this study are available in the main text.
